# The dual influence path of decent work perception on employee innovative behavior

**DOI:** 10.3389/fpsyg.2023.1302945

**Published:** 2023-12-21

**Authors:** Yan Yan, Di Deng, Yuqing Geng, Juan Gao, Enzhong Lin

**Affiliations:** ^1^Shanghai Dianji University, Shanghai, China; ^2^Guizhou University of Traditional Chinese Medicine, Guiyang, China; ^3^Chengdu Huizhixin Management Consulting Co., Ltd, Chengdu, China

**Keywords:** decent work perception, employee innovative behavior, job engagement, job burnout, authoritarian leadership

## Abstract

**Background:**

The goal of decent work (DW) is a win-win situation for both employees and employers. It promotes an individual’s employability and enhances the competitiveness of the organization.

**Design:**

Based on the conservation of resources theory (COR), this paper conducted survey on knowledge workers and analyzed the data by hierarchical linear model (HLM).

**Research purposes:**

This paper aims to examine how decent work perception (DWP) influences employee innovation behavior through the mediating effect of job engagement and burnout and the moderating effect of authoritarian leadership.

**Findings:**

Based on the results of statistical analyses conducted on 489 valid knowledge workers, it was demonstrated that DWP positively influence employee innovative behavior. Job engagement has a full mediating effect on the relationship between DWP and employee innovative behavior. The study did not support the mediating effect of job burnout, however. There is a positive moderating effect of authoritarian leadership on the relationship between DWP and job engagement and a negative moderating effect on the relationship between DWP and job burnout.

**Implications:**

In addition to contributing to theoretical studies on DW and work behavior, this paper also contributes to practice on employee motivation and leadership.

## 1 Introduction

In 1999, the International Labor Organization (ILO) proposed the concept of decent work (DW), which aimed to promote opportunities for women and men to obtain decent and productive employment under conditions of freedom, equity, security, and dignity. An organization committed to DW must ensure employees’ interests and develop their employability, actively promote competitive advantages for the organization (Elshater et al., 2022), and promote social sustainability (Geng and Maimaituerxun, 2022). As the organization’s most valuable asset, employees’ innovation behavior is a key source of innovation and competitive advantage. Thus, motivating knowledge workers’ innovative behavior has become increasingly crucial for individual employability, organizational innovation, and social advancement.

The existing research on DW can be divided into two research priorities. First, the macro-level of DW. The concept of DW originated in the field of economics and industrial relations. Researchers developed DW indexes at a macro level based on the ILO definition. Researchers evaluated and compared the status quo of DW in different countries and regions, including India, China, Nepal, Spain, and Brazil (Gil et al., 2008; Saha, 2009; Adhikari et al., 2012). Second is DW’s micro-level, also called decent work perception (DWP). The concept of DWP was derived from vocational psychology, organizational behavior, and human resource management. Using grounded theory and psychology of working theory from the individual perception perspective, researchers developed the dimensions and scales of DWP (Blustein et al., 2016; Douglass et al., 2017; Duffy et al., 2017; Ferraro et al., 2018; Seubert et al., 2021). Research has focused on the status quo of DW in various groups (Cruz et al., 2017), as well as the relationship between DW, work attitudes, and work behavior (Ferraro et al., 2020; McIlveen et al., 2021; Aybas et al., 2022; Xu et al., 2022). While studies on DW have been developing for more than 20 years, there are still some limitations. There is still room for improvement in the study of DW at the micro level. Despite a few DWP scales for employees, they lack validation in different contexts to ensure their universality. It is necessary to develop a scale of DWP for specific employees. The existing scale of DWP was developed for line workers. Knowledge workers differ from other workers regarding autonomy, motivation, and work behavior (El-Kassar et al., 2022; Hon et al., 2022). Thus, using current scales to measure knowledge workers’ DWP is inappropriate. More attention should be paid to the outcomes of DWP. In existing studies, DWP is considered an outcome variable and focuses on the realization of DW but disregards the impact of DWP as an antecedent variable on employee behavior.

Innovative behavior can be described as a process in which employees generate, develop, and implement new ideas to improve role performance (Janssen, 2000). Three aspects of employee innovative behavior have been discussed in previous literature. Individual factors, such as psychological capital and a proactive personality, influence innovative behavior (Sun and Huang, 2019; Alikaj et al., 2021). Second, organizational factors affect employee behavior through job characteristics, environmental factors, and leadership (Dedahanov et al., 2019; Qi et al., 2019; Lee et al., 2021). Furthermore, social factors, such as government policies and social networks, can influence innovative behavior (Lenihan et al., 2019). Only some empirical studies have examined innovation behaviors from a resource perspective in existing research. Individual and organizational factors influencing employee innovative behavior require a high level of resource input, according to the conservation of resources theory (COR). To achieve DW, one must satisfy an intrinsic human need for respect and self-value, promoting workplace innovation. From a resource perspective, it is therefore necessary to investigate the relationship between DW and employee innovative behavior.

Based on COR, the following study was conducted. (1) We tested how DWP effectively predicts employee innovative behavior. (2) We conducted empirical research on how DWP promotes employee innovative behavior through the mediating effect of job engagement driven by the resource-gain spiral. (3) We examined how DWP hinders employee innovative behavior due to the mediating effect of job burnout resulting from the resource-loss spiral. (4) We tested the moderating role of authoritarian leadership (AL) in the relationship between DWP, job engagement, and burnout.

Developing the empirical study between DWP and employee innovative behavior showed this study’s significance as follows. (1) The study makes an initial attempt to use DWP as the antecedent of employee innovation from the point of view of COR. (2) Research on the antecedents of employee innovative behavior thus shifts from an individual factor to an integration of contextual factors, raising social concern due to the deregulation of the labor market and its impact on workers’ well-being. (3) The study promotes DW studies by connecting DWP with work attitudes and behaviors and extending the scope of DW research.

## 2 Theoretical background

### 2.1 DW:Concept and measurement

DW was first proposed by Juan Somavia, ILO director, in 1999. The original definition is based on a sociological concept to promote opportunities for women and men to obtain decent and productive work in conditions of human dignity, security, and freedom. DW macro studies focus on the status of DW in a nation, country, or area. Using in-depth research, scholars from psychology and management introduced DW to vocational psychology and organizational behavior (Cooke et al., 2019). Therefore, DW has evolved into an interdisciplinary concept combining sociology, psychology, and management from a micro-level focusing on individual perceptions (Yan et al., 2023). As a result, DW at the micro level is also referred to as DWP. DWP studies emphasize individual self-value, which emphasizes the meaning and value of work (Di Fabio and Blustein, 2016), emphasizing that individuals can realize their self-value and dignity through work challenges.

The measurement of DW at the macro-level and micro-level has been developed since 1999. First, Ferraro et al. (2018) proposed a decent work questionnaire (DWQ) comprising 31 indexes related to the four pillars of human rights, employment equity, social dialogue, and social protection. This measurement is based on a macro-level definition of DW. In addition, Duffy et al. (2017) have developed a decent work scale (DWS) based on the psychology of working theory at a micro level. DWS comprises five dimensions: safe working conditions, access to health care, adequate compensation, free time and rest, and organizational value. Lastly, Yan et al. (2023) developed a decent work perception scale (DWPS) for knowledge workers from a micro-level perspective. This scale is designed from grounded theory, containing 4 dimensions and 13 items. Job security, respect and support, self-value, and professional skills are four dimensions.

In addition to the above measurements, other scholars have developed DW measurement tools from both macro and micro perspectives in recent years. For instance, 9 indicators testing security, farming, and hospitality industries in South Africa (Webster et al., 2015), 8 factors for employees from the hospitality sector (Garcia-Rodriguez et al., 2021), and decent work index (DWI) testing in Gauteng City-Region (Mackett, 2017). The prevalence of these measurement tools suggests that research on DW is increasingly focused on studies of perception at the micro-level. The research focus shifted from the macro-level to the micro-level because different countries and nations may have various social security and social protection systems. Scholars have difficulty standardizing statistical indexes for macro-level indicators across different countries. However, micro-level scales from individual perception are self-reported, which avoids this obstacle.

Even though DW research has been developed for more than 20 years, there are still some limitations in the current research. The first limitation is the lack of a consistent DWP scale. Scholars have conducted extensive research on the micro-level of DW, but there has yet to be a consensus on the definition of DWP at the micro-level. The lack of a consistent definition as a basis for DWP tools development at the micro level has resulted in the need for uniformity in the measurement tools for DWP. Second, the structural dimensions of DWP vary from group to group due to the differences in job demands among workers engaged in different types of work.

### 2.2 Conservation of resource theory

This study aims to fill the research gap by applying COR to DW research to investigate the relationship between DWP and individual behavior. DWP studies are theoretically based on self-determination theory, social exchange theory, broaden-and-build theory, and psychology of working theory (Huang and Yuan, 2022). Based on the existing theoretical foundations, scholars have studied DWP’s antecedent and outcome variables, enriching the research results of DW. According to COR, an individual’s attitude and behavior are affected by DWP dual influence paths from the perspective of a resource gain spiral and a resource loss spiral. Individuals tend to preserve, protect, and acquire their resources, leading to different behaviors based on resource gains and losses (Hobfoll et al., 2018). Therefore, it is necessary to introduce COR into the DWP study. One significant advantage of COR is that it can more clearly describe DWP’s resource gain and loss dual paths on individual behavior. Further, COR can provide multiple potential factors that may influence the relationship between DWP and subsequent outcomes at the same time. To put it another way, COR can facilitate a deeper understanding of the mechanisms by which DWP affects work-related outcomes, including moderators and mediators.

According to COR, individuals who value a particular resource are more likely to reinvest that resource, acquire new resources, and engage in behaviors that benefit them and their organizations (Hobfoll et al., 2018). A critical resource for employees is job engagement. Job engagement means that “A person who is involved in his work takes his job and career seriously, has meaningful values and components of his identity, will be affected emotionally and significantly by work experience, and will be mentally preoccupied with work” (Jans, 1982, p. 57). Examining job engagement is critical. Job engagement, or a positive attitude in the workplace, is essential in improving employee behavior and a source of competitive advantage for the organization (Hu et al., 2023). We thus consider job engagement as the mediator in the relationship between DWP and employee innovative behavior.

COR suggests that individuals are prone to stress when faced with resource loss or the threat of resource loss. People who lack resources are more likely to lose existing resources and more likely to lose even more resources under stress pressure, thus creating a cycle of resource loss (Hobfoll et al., 2018). Another vital resource for employees is job burnout. Job burnout is “a prolonged response to chronic emotional and interpersonal stressors on the job and is defined here by the three dimensions of exhaustion, cynicism, and a sense of inefficacy” (Maslach, 2003, p189). The fatigue and burnout of employees inhibit their ability to be innovative due to stress and a lack of time, resources, and support (Kwon and Kim, 2020). As a result, this study examines the role of burnout as a mediator between DWP and employee innovative behavior.

In addition, it is crucial to identify the boundary conditions within which DWP can influence workplace outcomes. As organizations increasingly utilize workgroups (Ohana and Stinglhamber, 2019), team leaders (supervisors) have acquired unprecedented influence over work teams (Wei et al., 2022). However, it remains to be seen how leaders may influence the consequences of DWP. As a dimension of paternalistic leadership, AL is characterized by high control over subordinates (Chiang et al., 2021). Authoritarian leaders use their authority, which is enshrined in organizational hierarchies, to demand absolute obedience from their subordinates (Pizzolitto et al., 2023). It has been recognized as a universal phenomenon in the Chinese working environment (Zhang et al., 2021). Most studies have examined the direct effects of AL at the workplace rather than its potential moderating effects (Yang and Wei, 2018). AL has been found to hinder team members’ ability to obtain job resources and result in the loss of personal resources (Asim et al., 2021). According to COR, employees may cope with losing resources through AL by reallocating other available resources, such as those derived from DWP. As a result, AL may reduce the influence of the DWP on employee innovative behavior by misappropriating job-related resources obtained from the DWP.

## 3 Hypothesis

### 3.1 Main effect between DWP and employee innovative behavior

We propose that DW has a direct impact on employee innovative behavior. DWP reflects an individual’s perception of work based on personal needs and comparison with others (Qing et al., 2016). A qualitative research result, combined with exploratory factor analysis and confirmative factor analysis for knowledge workers, shows that DWP includes job security, respect and support, self-value, and professional skills (Yan et al., 2023). Job security is related to employees’ perceptions of income, benefits, and work safety provided by the employer. An employee’s perception of fairness at work and the respect and support they receive from their colleagues and leaders are included in respect and support. Self-value refers to employees’ perceptions of freedom, autonomy, and values in the work process. Knowledge and skills applied to the challenges of the job are referred to as professional skills.

Using the COR model, we consider job security and professional skills to be direct resources, whereas respect & support, and self-value are indirect resources. Research has demonstrated that job insecurity contributes significantly to employee stress and resource loss. Therefore, securing income and ensuring the safety of employees is essential for preserving and acquiring resources. Another vital resource is employees’ sense of control over their work due to their professional skills and challenging jobs (Kakavand et al., 2019). Employees with greater direct access to resources typically report a strong sense of job security and high levels of expertise, which can reduce stress on the job by providing them with a sense of psychological security (Newman et al., 2019). Respect, support, and self-value are indirect resources that come from appreciating and recognizing an employee’s commitment to work, which is a powerful psychological motivator for the employee. As a result, employees with more indirect resources are more likely to have their ideas recognized and realized at work, which can provide psychological incentives for employees to work in an innovative manner.

It has been shown that knowledge workers satisfy their job demands by applying their job resources (Schaufeli and Bakker, 2004). Employees’ innovative behavior may be significantly influenced by their ability to meet job demands (Li and Hsu, 2016). Employees with higher DWP have access to more direct and indirect resources, enabling them to use resources to meet job demands. Fulfilling job demands through the efficient utilization of resources creates psychological incentives that motivate employees to acquire more resources, thus entering the value-gain spiral. By accumulating and gaining resources, employees will continue to develop new ideas and innovate their working methods. Consequently, their innovative behavior can be enhanced and promoted. So, we propose that:

*H1*: DWP has a significant positive effect on employee innovation behavior.

### 3.2 Mediating role of job engagement

It is well known that burnout and engagement are polarized aspects of work well-being (Schaufeli et al., 2002). A negative state known as burnout occurs when an individual’s dedication to a career does not yield the desired results (Bakker et al., 2014). In contrast to work burnout, job engagement describes a positive integration between self and work achieved through self-control (Kahn, 1990). Job engagement includes vigor, dedication, and absorption (Schaufeli et al., 2002). A vigorous individual is energetic, possesses a high level of mental resilience at work, and is willing to work hard and persist despite obstacles. Dedication is characterized by high involvement in one’s work, a sense of passion, inspiration, pride, and challenge. Absorption is defined as total concentration and attention, feeling that time passes rapidly at work and not wanting to stop working.

Based on COR, we propose that job engagement mediates DWP and employee innovation behavior. It is common for knowledge workers to have a high education level, engage in innovative work rather than repetitive and monotonous tasks, and exercise a significant degree of autonomy at work. The characteristics of knowledge work suggest that innovation is required at work. Following COR, knowledge workers’ perception of DW is determined by their capacity to meet the demands of their jobs (Huang and Yuan, 2022). Workers with high DWP are more likely to have access to resources, such as a secure job, strong professional skills, support and respect from their organizations, as well as a high sense of self-value. Previous findings on Brazilian physicians suggest a correlation between DW and job engagement (Ferraro et al., 2020). By using resources effectively, knowledge workers can rationally assess individual resources when there is a high demand for innovation. In the face of difficulties, knowledge workers are more engaged in their tasks and are more willing to share their knowledge with others to overcome those challenges (Wu and Lee, 2020). Positive emotions have been found to stimulate creative inspiration and innovative behavior in knowledge workers (Montani et al., 2020). An employee whose innovative behavior is appreciated by the organization receives more intrinsic and extrinsic rewards, accumulating personal resources. The knowledge worker with a high DWP may be able to accumulate resources through engagement, enter a gain-spiral of resources, and demonstrate innovative behavior as a result. We propose that:

*H2*: Job engagement mediates DWP and employee innovation behavior.

### 3.3 Mediating role of job burnout

We propose that job burnout mediates the relationship between DWP and employee innovative behavior. As the opposing facet of work well-being, burnout encompasses emotional exhaustion, cynicism, and low professional efficacy (Greenglass et al., 2001). An emotionally exhausted individual is not impulsive toward work and feels frustrated, stressed, and even frightened of it. A cynical employee keeps a distance from his or her coworkers and does not show enthusiasm or commitment to the work he or she does. Low professional efficacy refers to individuals who have a negative view of themselves and believe they are ineffective in their jobs.

COR suggests that when knowledge workers are faced with innovative requirements, they should utilize the existing resources to accomplish the task. Knowledge workers with a low DWP have fewer resources, unsecured employment, fewer skills, less organizational respect, and lower self-value at work. Lack of resources can cause psychological stress when faced with high job demands. Constant stress and tension produce burnout (Yui et al., 2021). In other words, job burnout contributes to negative emotions among knowledge workers, hinders their personal development and goal attainment, and inhibits their creativity (Li et al., 2019). A loss spiral occurs when individuals lose the resources they already possess and fail to acquire new resources, leading to further loss. Knowledge workers with a low DWP may exhibit negative emotions due to high job demands, including anxiety due to a lack of resources. Burnout can be caused by negative emotions, preventing access to resources, and inhibiting the generation of new ideas. Therefore, we propose that:

*H3*: Job burnout mediates DWP and employee innovation behavior.

### 3.4 Moderating effects of authoritarianism leadership

From a leadership perspective, AL emphasizes the absolute authority of the leader. A leader closely monitors and manages subordinates who exhibit behaviors of attachment and obedience (Pizzolitto et al., 2023). Based on COR, leadership styles may affect a subordinate’s access to resources at work (Mahmood et al., 2020). Through positive leadership styles, subordinates are led into a resource-gain spiral by receiving positive feedback, which in turn facilitates the acquisition of additional resources by the subordinate. On the other hand, an adverse leadership style can result in negative feedback for subordinates, leading them to diminish their resources and resulting in a spiral of resource loss. Authoritative leaders demand high standards of work results from their subordinates, tightly control the work process, and often criticize and nitpick their work. Undoubtedly, this leadership creates negative feedback to the subordinates, which may result in psychological stress. Research findings suggest that AL can have a negative impact on subordinates’ job satisfaction and organizational embeddedness (Siddique et al., 2020).

Knowledge workers with a high DWP have more resources, such as more robust job security, professional skills, respect and recognition, and a higher sense of self-value. Abundant resources motivate employees to increase job engagement (Zhang et al., 2019). A leader who exhibits AL behaviors will monopolize power, undermine the autonomy of his or her subordinates, disregard their opinions and suggestions, and frequently criticize and reprimand them. Subordinate support is undermined by this leadership behavior, resulting in weaker job resources for the subordinate. Furthermore, AL may lead to negative feelings among subordinates, who may feel that their leaders are more demanding and have less autonomy in their work. Negative emotions can diminish an employee’s commitment to work and result in resource loss. If resources are depleted, fewer resources are available to acquire new ones, leading to psychological stress and low employee motivation (Hobfoll et al., 2018). Previous research on Chinese workers in Taiwan has shown that subordinates experience declines in motivation and job engagement when their leaders exhibit AL behaviors (Shu, 2015). Thus, knowledge workers with high DWP may experience work resource loss when under AL, resulting in low work engagement. Therefore, we propose that:

*H4*: AL has a significant negative moderating effect on the relationship between DWP and job engagement.

Knowledge workers with a low level of DWP have fewer resources available at work, lack adequate job security, possess fewer professional skills, and receive less recognition and respect at work, making it more difficult to realize their self-value in the workplace. Knowledge workers who have a low level of DWP cannot meet the high job demands due to a lack of job resources when faced with AL. As a result, they tend to exhibit negative emotions at work, like procrastinating, being negligent, and not completing errands efficiently. Anxiety and boredom may result from long-term negative emotions, leading to job burnout. Anxious employees will likely invest less time, energy, and emotion into the job. As a result of mis-utilizing the resources, knowledge workers may lose the resources they already possess. A lack of resource investment may accelerate the speed at which resources are lost, resulting in continuous resource depletion, and thus entering a spiral of resource losses. Therefore, under AL, subordinates with low DWP show more significant levels of burnout due to a lack of resources. Therefore, we propose that:

*H5*: AL has a significant positive moderating effect on the relationship between DWP and job burnout.

**Figure 1:**
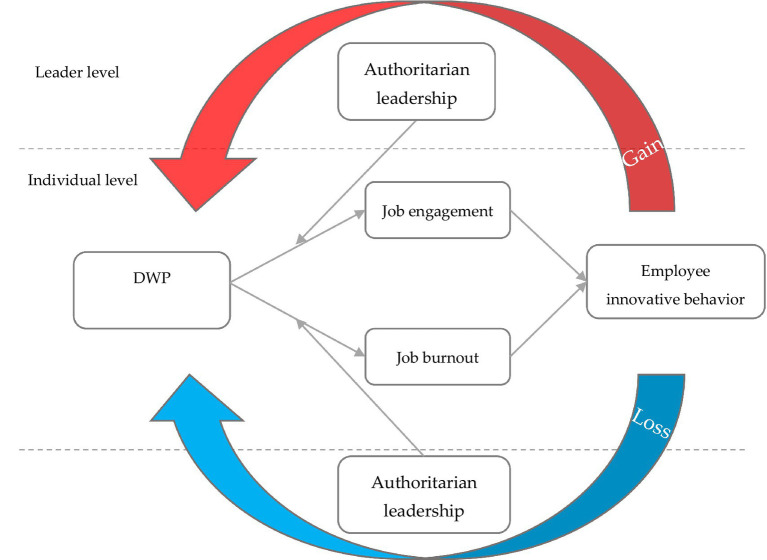
Theoretical framework.

The theoretical framework of DWP, job engagement, job burnout, AL and employee innovative behavior is shown below (see Figure 1).

## 4 Methods

### 4.1 Procedures and samples

This study focuses on knowledge workers. The distribution of knowledge workers is characterized by industry aggregation, regional aggregation, and differences in ownership of enterprises. Currently, Chinese enterprises are characterized by differentiated development across industries, unbalanced development across regions, and diversified ownership. We used a stratified convenience sampling method to meet the requirement of a diverse sample. First, we targeted industries that gather knowledge workers, such as banking, securities, investment, insurance, TIC (testing, inspection, and certification), colleges and universities, and research laboratories. Second, we selected economically developed regions for sampling, such as metropolitan areas such as Beijing, Shanghai, and Guangzhou, as well as provincial capitals of the second tier. Our final step was to choose samples from different types of organizations, including state-owned enterprises, private enterprises, foreign enterprises, public institutions, and governments. The paper questionnaire was distributed face-to-face to ensure a high rate of return.

This survey was approved by the Academic Ethics Committee of the School of Business in July 2022. Subjects will receive a written consent form, which they must read and sign before starting the survey. We promised that the study would be conducted anonymously, and all data would be used for academic research only and not for commercial purposes. The subjects were informed that their research data would be stored on an encrypted computer and not made available to the public. The survey was conducted over four months, from September 1st, 2022, to December 31st, 2022. We distributed 580 questionnaires and collected 489 valid questionnaires with a valid recovery rate of 84.31%. The descriptive statistics of the sample are shown in Table 1. Males accounted for 40.5% of the sample, while females accounted for 59.5%, slightly higher than males. The age distribution of the sample indicates that 7.6% are 25 years of age or younger, 55.8% are 26–34 years of age, 27.2% are 36–45 years of age, and 9.4% are 46 years of age or older. This is in line with the characteristics of the human capital curve. As for education level, 9.2% of respondents hold a junior college degree or lower, 65% hold an undergraduate degree, 18.6% hold a postgraduate degree, and 7.2% hold a doctoral degree. As for ownership, state-owned enterprises accounted for 59.1%, private enterprises accounted for 10%, foreign enterprises accounted for 3.7%, public institutions and government accounted for 24.7%, and others accounted for 2.5%. Banking accounted for 28% of the industry distribution; insurance accounted for 37%; securities and investment accounted for 4.9%; TIC accounted for 6.1%; and colleges, universities, and research institutions accounted for 22.5%, reflecting the clustering characteristics of knowledge workers within the industry. In terms of organization tenure, 6.1% of samples work for half a year or less, 5.7% work for 0.6–1 year, 22.5% work for 1.1–3 years, 19.2% work for 3.1–5 years, 19% work for 5.1–10 years, and 27.4% work for 10.1 years and above. Accordingly, knowledge workers prefer stable employment and will likely remain employed for a lifetime.

**Table 1:** Descriptive statistical analysis of formal survey.

Variable	Item	Percentage (%)
Gender	Male	40.5
	Female	59.5
Age	25 years old and below	7.6
	26–35 years old	55.8
	36–45 years old	27.2
	46 years old and above	9.4
Education	Junior college and below	9.2
	Undergraduate college	65.0
	Postgraduate college	18.6
	PhD	7.2
Ownership	State-owned business	59.1
	Private business	10.0
	Foreign enterprise	3.7
	Public organizations and government	24.7
	Others	2.5
Industry	Banking	28.0
	Insurance	37.0
	Security	4.9
	TIC	6.1
	Public organizations and governments	22.5
	Others	1.4
Organization tenure	0.5 year and below	6.1
	0.6–1 year	5.7
	1.1–3 years	22.5
	3.1-5 years	19.2
	5.1–10 years	19.0
	over 10 years	27.4

### 4.2 Measures

In this study, we used the newly developed scale for knowledge workers, DWPS. It was found that the DWPS had good reliability and validity among Chinese knowledge workers. We utilized the Chinese version of scales for job engagement, job burnout, and AL and the original English scale for employee innovative behavior. We performed back-translation to ensure the validity of the employee innovative behavior scale. To begin with, a doctoral student in human resource management translated the English version of the employee innovative behavior scale into Chinese. The Chinese text was then translated into English by another human resource management PhD student. A professor of human resource management compared the two English versions of the scale and checked items in the Chinese version.

The questionnaire was divided into leaders’ and subordinates’ parts. Leaders and subordinates needed to be rigorously surveyed in one-to-one pairings to satisfy the moderating effect between leaders and subordinates designed for this study. The questionnaire was divided into three parts. Part one was guidance, including the study purpose and research content. Part two was the measurement items for each variable. All of them were on a 5-point Likert scale, with 1 indicating strongly disagree, 2 indicating disagree, 3 suggesting neutrality, 4 showing agree, and 5 indicating strongly agree. Part three was demographic variables.

We conducted a reliability test, validity test, and common method bias test for the formal survey. We used reliability and KMO tests to ensure the scale’s reliability and validity (see Table 2). It is required to take a common method bias test since the survey was self-reported. The Harman single-factor test was used to test respondents’ self-reports of DWP, AL, job engagement, and job burnout based on the criteria provided by Podsakoff et al. (2003). We did an exploratory factor analysis of all the variables and extracted seven unrotated factors with eigenvalues greater than 1 by principal component analysis. Results showed that the explained variance ratio of the first factor was 29.099%. Referring to Podsakoff et al. (2003), it was considered that there was no serious common method bias among the variables since the explained variance ratio of the first factor was lower than 40%.

**Table 2:** Reliability test results and validity test results of formal survey.

Variable	Dimension	Cronbach’α	KMO	Explained variance
DWP	Job security	0.842	0.931	64.788
	Professional skills	0.876		
	Respect & support	0.869		
	Self-value	0.857		
Job burnout	Emotional exhaustion	0.906	0.905	71.742
	Cynicism	0.899		
	Low professional efficacy	0.855		
Job engagement		0.901	0.747	83.714
AL		0.934	0.907	65.778
Employee innovative behavior		0.774	0.775	59.903

#### 4.2.1 DWP

We selected the DWPS developed by Yan et al. (2023) for knowledge workers as the measurement tool for DW. This scale contains 14 questions in four dimensions: job security, professional skills, respect & support, and self-value. As shown in Table 2, the Cronbach’α value of each dimension for DWPS is over 0.8, with 64.788% of the explained variance ratio, indicating high reliability and validity. We do not revise the questions since they were developed for knowledge workers, which is adequate for our research subjects. The complete scale of DWPS can be found in the Table A1 in the Appendix.

#### 4.2.2 Job engagement

This study used the Utrecht Work Engagement Scale (UWES) developed by Schaufeli et al. (2019), which includes dimensions such as vigor, dedication, and absorption. The Cronbach’α value of UWES is 0.901, more significant than 0.7, demonstrating good reliability. The KMO value is 0.747, and the variance explained is 83.714%, which suggests good validity.

#### 4.2.3 Job burnout

We use the Burnout Inventory General Survey (MBI-GS) developed by Maslach et al. (2001) to measure job burnout. The MBI-GS contains 16 questions in three dimensions: emotional exhaustion, cynicism, and low professional efficacy. The Cronbach’α values for each dimension are 0.906, 0.899, and 0.855, respectively, indicating good reliability. The KMO value is 0.905, and the variance explained is 71.742%, suggesting good validity.

#### 4.2.4 Authoritarian leadership

This study utilized the authoritarian leadership scale (ALS) developed by Wu and Zhao (2009) as the measurement tool. Conducting on the definition of AL by Cheng et al. (2000), Wu and Zhao (2009) developed ALS in the Chinese context. The ALS consists of nine questions using subordinates’ ratings of their superiors’ leadership style. The Cronbach’α of ALS is 0.934, which indicates that the scale has good reliability. The KMO value is 0.907 and the variance explained ratio is 65.778%, which demonstrates the questionnaire has good validity.

#### 4.2.5 Employee innovative behavior

We adopt the innovative behavior scale developed by De Jong and Den Hartog (2010), which measures employee innovative behavior from a process perspective. This scale contains four dimensions: idea development, idea generation, idea dissemination, and idea implementation. Based on the leader’s evaluation, this scale is shown to be an effective tool for measuring the innovative behavior of knowledge workers. The Cronbach’α value of the scale is 0.774, indicating good reliability. The KMO value is 0.775, and the variance explained is 59.903%, suggesting good validity.

#### 4.2.6 Control variables

We choose gender (1 = male, 2 = female), age (1 = 25 years old and below, 2 = 26–35, 3 = 36–45, 4 = 46 years and above), education (1 = junior college and below, 2 = undergraduate college, 3 = postgraduate college, 4 = PhD), organization ownership (1 = state-owned business, 2 = private business, 3 = foreign enterprise, 4 = public organizations and governments, 5 = others), industry (1 = banking, 2 = insurance, 3 = security, 4 = TIC, 5 = public organizations and governments, 6 = others) and organization tenure (1 = 0.5 year and below, 2 = 0.6–1 year, 3 = 1.1–3 years, 4 = 3.1-5 years, 5 = 5.1–10 years, 6 = over 10 years) as control variables. Organization tenure is calculated as the years since the respondent works in this organization.

## 5 Results

### 5.1 Correlations and ANOVA

Correlation analysis, as the primary test for the relationship between variables, predicates the hypothesis. We conducted a correlation analysis on five variables, and the results are shown in Table 3. DWP is positively correlated with job engagement (r = 0.681), negatively correlated with job burnout (*r* = −0.531), and positively correlated with employee innovative behavior (*r* = 0.221). AL is negatively correlated with job engagement (*r* = −0.134) and positively correlated with job burnout (0.439). Job engagement is positively correlated with employee innovative behavior (*r* = 0.233). The correlation results indicate the relationship between the five variables in the hypothesis and their direction.

**Table 3:** Correlation analysis results.

Variable	M	SD	1	2	3	4	5
1. DWP	3.881	0.562	1				
2. Job engagement	2.893	0.836	0.681**	1			
3. Job burnout	2.428	0.550	−0.531**	−0.491**	1		
4. AL	2.634	0.773	−0.172**	−0.134**	0.439**	1	
5. Employee innovative behavior	3.148	0.391	0.222**	0.233**	−0.038**	0.021	1

**p < 0.01; *p < 0.05.

Group t-tests and ANOVA are conducted to test whether demographic factors affect the relationship between independent and dependent variables. We utilize the group t-test to examine gender differences. Results of the F-test on the two genders show that the *p*-values of all factors are more significant than 0.05 at the significance level of 5%, indicating no significance in all factors between the two genders. We conducted ANOVA on age, education, ownership, industry, and organization tenure. Education, ownership, industry, and organizational tenure significantly affect the relationship between independent and dependent variables, while age does not play a significant role. As a result, we need to control the effect of education, ownership, industry, and organization tenure on variables when testing the hypothesis.

### 5.2 Hypothesis test

We hierarchical linear modeling (HLM) to examine the moderating effect by using statistical software HLM6.08. HLM is suitable for empirical analysis of nested relationships, which means that the observed data belong to different levels. In this study, a team-level variable, AL refers to leaders’ behavior to monitor and control their subordinates. Individual-level variables include DWP, job engagement, job burnout, and employee innovation behavior. We use the HLM to examine nested data at the team and individual levels to obtain accurate and reliable results.

#### 5.2.1 Main effects

H1 proposes that DWP positively affects employee innovative behavior. Team leaders evaluate innovative behavior, and subordinates self-report DWP. The team data and individual data are nested. Therefore, it is necessary to conduct HLM to test the relationship between DWP and employee innovative behavior. Table 4 shows a positive correlation between DWP and employee innovative behavior (M3, *y* = 0.088, *p* < 0.01). Therefore, H1 is supported.

**Table 4:** HLM results of H1.

Variable	Employee innovative behavior						
	M1	M2	M3	M4	M5	M6	M7
Constant	3.144	3.145	3.137	3.144	3.145	3.144	3.144
Education		0.005	0.008	0.004	0.008	0.005	0.008
Ownership		0.021	0.022	0.019	0.019	0.022	0.020
Industry		0.002	0.001	0.001	0.000	0.002	0.0002
Organization tenure		−0.010	−0.008	−0.009	−0.008	−0.006	−0.007
Team size		−0.008	0.015	0.011	0.008	0.012	0.012
DWP			0.088**				
Deviance	330.098	355.731	352.305	354.9	357.58	356.115	359.979

**p < 0.01; *p < 0.05.

#### 5.2.2 Mediating effects

H2 proposes that job engagement mediates the relationship between DWP and employee innovative behavior. H3 supposes job burnout mediates the relationship between DWP and employee innovative behavior. We utilize HLM to test the hypothesis since the employee innovative behavior from the team level and job engagement, job burnout, and DWP from the individual level are nested data. According to Baron and Kenny (1986), we analyze mediating effects in two steps. Step 1 is to test the positive effect of DWP on employee innovative behavior. Step 2 is to examine the effect of job engagement and job burnout on employee innovative behavior when controlling the effect of education, ownership, industry, and organization tenure.

Table 5 shows that job engagement has a significant effect on employee innovative behavior (γ60 = 0.048, *p* < 0.05) in step 2, while job burnout does not have a significant effect on employee innovative behavior (γ70 = 0.031, *p* > 0.1). The results indicate that job engagement mediates the relationship between DWP and employee innovative behavior. Therefore, H2 is supported. Job burnout does not mediate between variables, and H3 is not supported. As shown in Table 5, DWP does not significantly affect employee innovative behavior in step 2 (γ50 = 0.043, *p* > 0.1), indicating that job engagement has full mediation between DWP and employee innovative behavior.

**Table 5:** HLM results of H2 and H3.

Variable	Employee innovative behavior		
	Step 1	Step 2	
Constant (γ _00_)	3.137	3.136	3.144
Education (γ_10_)	0.008	0.011	0.007
Ownership (γ_20_)	0.021	0.02	0.021
Industry (γ_30_)	0.0006	0.004	0.0001
Organization tenure (γ_40_)	−0.008	−0.01	−0.01
DWP (γ_50_)	0.088**	0.043	0.102**
Job engagement (γ_60_)		0.048*	
Job burnout (γ_70_)			0.031
Team size (γ_01_)	0.015	0.0168	0.012
Deviance	352.305	352.185	356.438

**p < 0.01; *p < 0.05.

#### 5.2.3 Moderating effects

**Figure 2:**
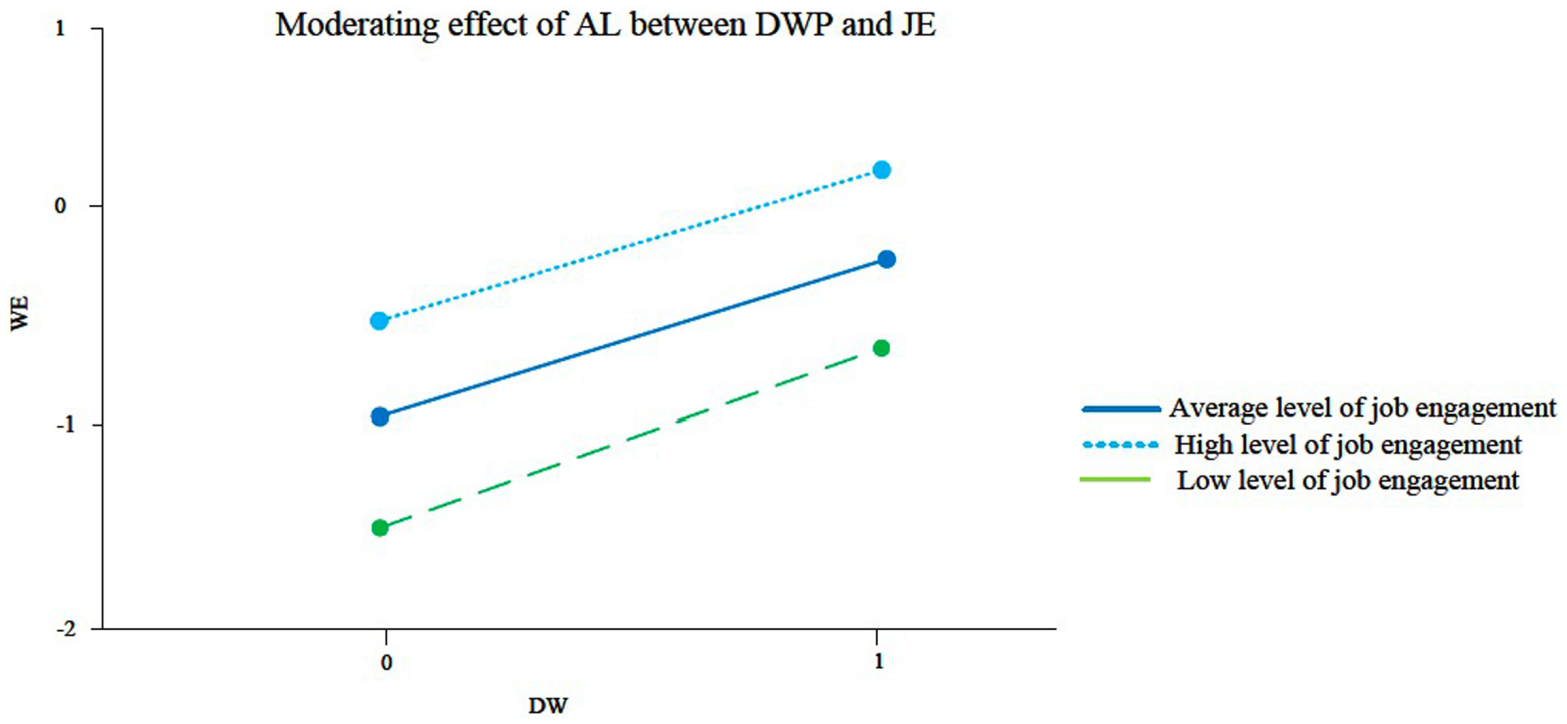
Moderating effect of AL between DWP and JE.

H4 proposes that AL has a negative moderating effect on the relationship between DWP and job engagement. Since AL is a team-level variable while DWP and job engagement are individual-level variables, we need to construct the HLM to analyze the moderating effect of AL. The interaction of DWP and AL in Table 6 is shown as DWP*AL, which has a significant negative effect on job engagement (γ70 = −0.175, *p* < 0.01, See Figure 2). Thus, H4 is supported.

**Table 6:** HLM result of H4.

Variable	Job engagement			
	M1	M2	M3	M4
Constant (γ_00_)	2.904	2.914	2.900	2.903
Education (γ_10_)		−0.052	−0.055	−0.061
Ownership (γ_20_)		0.052	0.040	0.041
Industry (γ_30_)		−0.035	−0.049	−0.052
Organization tenure (γ_40_)		−0.028	0.003	0.011
DWP (γ_50_)			0.983**	0.997
AL (γ_60_)			−0.033	−0.001
DWP*AL (γ_70_)				−0.175**
Team size (γ_01_)		−0.059	−0.023	−0.029
Deviance	1169.481	1185.67	931.682	927.361

**p < 0.01; *p < 0.05.

**Figure 3:**
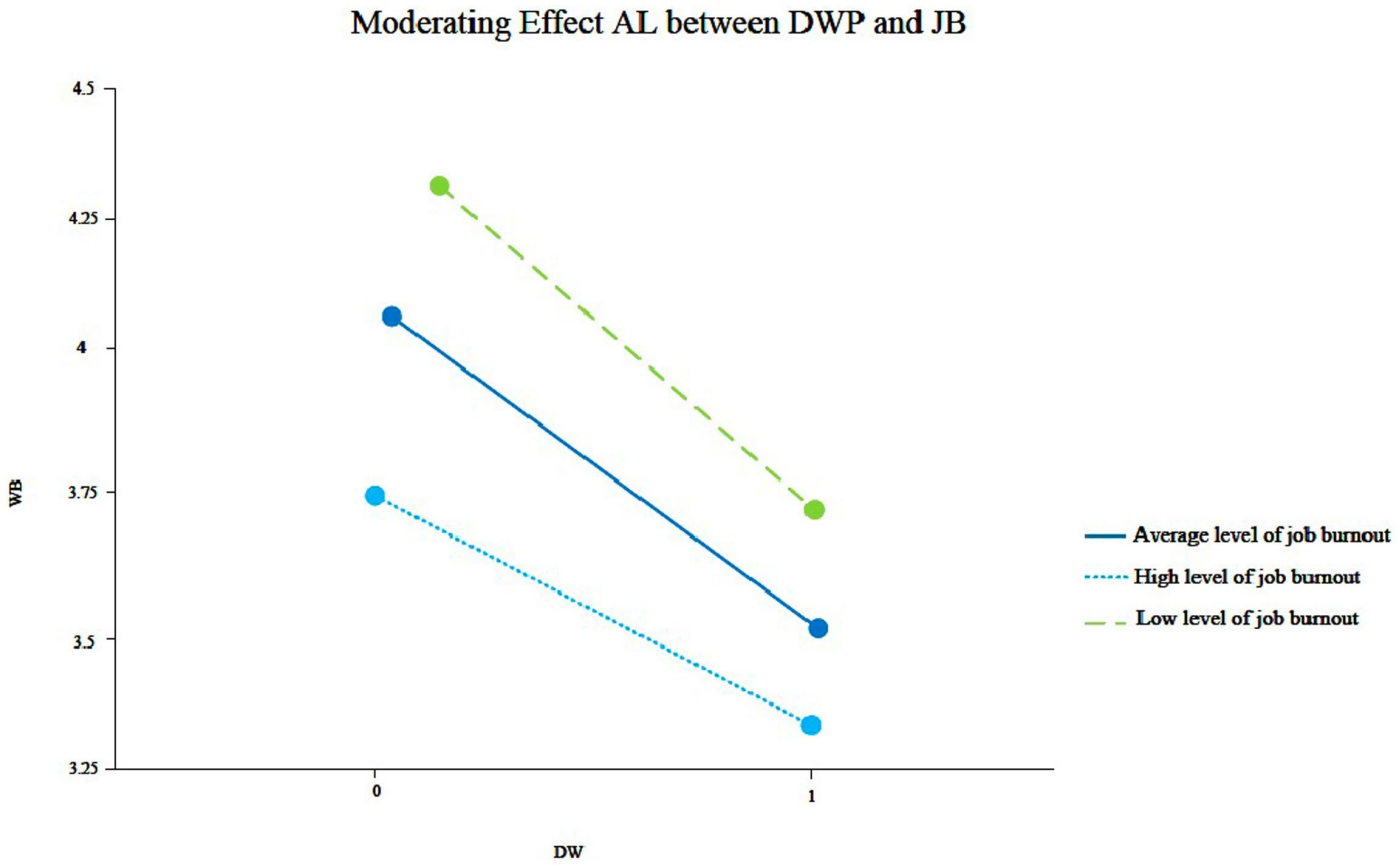
Moderating effect of AL between DWP and JB.

H5 proposes that AL has a positive moderating effect on the relationship between DWP and job burnout. We used the same steps as H4, and the study results are shown in Table 7 and Figure 3. The interaction between DWP and AL has a significant positive effect on job burnout (γ70 = 0.165, *p* < 0.01, See Figure 3). Therefore, H5 is supported.

**Table 7:** HLM result of H5.

Variable	Job burnout			
	M1	M2	M3	M4
Constant (γ_00_)	2.425	2.423	2.424	2.423
Education (γ_10_)		0.006	0.017	0.023
Ownership (γ_20_)		−0.0153	−0.017	−0.017
Industry (γ_30_)		−0.028	0.006	0.009
Organization tenure (γ_40_)		0.054**	0.023	0.016
DWP (γ_50_)			−0.4535**	−0.467**
AL (γ_60_)			0.240**	0.213**
DWP*AL (γ_70_)				0.165**
Team size (γ_01_)		0.032	0.011	0.016
Deviance	766.468	781.288	581.259	569.926

**p < 0.01; *p < 0.05.

## 6 Discussion

### 6.1 Key conclusion

This study confirms that knowledge workers’ DWP significantly positively affects employee innovative behavior (H1). Job engagement has a fully mediating effect on DWP and innovative behavior (H2). However, this study did not support the mediating effect of job burnout on DWP and innovative behavior (H3). AL negatively moderated the relationship between DWP and job engagement (H4) and positively moderated the relationship between DWP and job burnout (H5). Job engagement tends to decline under AL when knowledge workers have high DWP. Knowledge workers tend to show high levels of burnout with low DWP. Knowledge workers, however, tend to experience higher levels of burnout under the influence of AL.

### 6.2 Theoretical contributions

The main effect of H1, DWP has a positive effect on employee innovative behavior and expands the research on the impact of DWP on employee behavior. In recent years, with the development of individual DW scales, scholars have begun to explore the outcome variables of DWP. It was found that DW significantly positively affects employees’ in-role performance, organizational citizenship behavior (Huang and Yuan, 2022), proactive behavior, and voice behavior (Sheng and Zhou, 2022). However, there needs to be more research into the relationship between DW and employee innovation behavior. This study expands the scope of the relationship between DWP and employee behavior. It contributes to the basis for the mechanism of DWP and employee innovative behavior.

H2 and H3 further explore the relationship between job engagement and job burnout. This study verified the fully mediating role of job engagement between knowledge workers’ DWP and innovative behavior. However, it did not validate job burnout’s mediating role. This may be because innovative behavior, as a positive behavioral outcome, is more likely to be influenced by positive attitudes (Kwon and Kim, 2020). The findings of this study again validate that job engagement, as a positive emotion, is the opposite of job burnout, which is a negative emotion. Previous research has found that job engagement can protect against job burnout (Maslach and Leiter, 2016). Employees who do not engage in their jobs do not necessarily mean they are burnout (Conway et al., 2016). Therefore, job engagement and burnout may have a non-linear or U-shaped relationship. There is a complex and diverse relationship between engagement and burnout, indicating the need for further research. Knowledge workers have fewer simple and repetitive tasks as a result of the nature of their work. Their work processes must be improved, more intellectual intelligence must be consumed, and more creative ideas must be generated. This job requires a high level of engagement and is less likely to lead to burnout. This may be one of the reasons burnout is not significant. Researchers can explore the relationship between engagement and burnout in other groups, such as industrial workers and informal employees, in future studies.

H4 and H5 contribute to DW boundary conditions. Leadership is the team-level variable, while DWP is the individual-level variable. This study introduces AL, a team-level variable, to the individual-level study of DWP using HLM. It provides new ideas for cross-level research on DW based on the results of this study. Previous studies have confirmed the impact of positive leadership on DW, such as entrepreneurial leadership (Shehri et al., 2022), and negative leadership styles, such as toxic leadership (Casqueira, 2019). However, leaders’ leadership is diverse and complex. Exploring the impact of leadership on DW in different cultural contexts is conducive to boundary studies of DW. AL is a unique style of leadership within the Chinese cultural context. Therefore, this study not only helps scholars to understand the role of contextual factors in the relationship between leadership and individual attitudes and behaviors but also facilitates scholars to explore the boundaries of leadership on DW in various cultural contexts in the future.

### 6.3 Practical implications

This paper provides new insights into how to develop the innovative behavior of knowledge workers. On the one hand, we found that DWP had a significant positive effect on employee innovative behavior. Knowledge workers are vital in enhancing the organization’s competitive advantage as vital capital. Knowledge workers’ DW includes job security, respect and support, self-value, and professional skills. The research results can guide managers in developing innovative behavior within their employees by providing them with a secure job, respecting their efforts, recognizing their performance, and improving their professionalism. Additionally, we found that job engagement mediates the relationship between DWP and employee innovation. Monitoring employee engagement can assist managers in predicting innovative performance among employees.

The results of this study provide new insights into how leadership can be transformed. We found that AL negatively moderates the relationship between DWP and job engagement and positively moderates the relationship between DW and job burnout. As a result of their traditional Chinese cultural background, Chinese leaders tend to demonstrate AL behaviors. The growing group of new generation Y employees has increasingly become the main force of China’s organizations. The majority of them are the only child in their family. Parents give excessive care and love. At work, they are eager to maintain equal communication with their supervisors. The AL style with large power distances is not preferred by new generations, inhibiting the development of innovative behavior. Leaders should change their AL style to manage the new generation of knowledge workers.

### 6.4 Limitations and future directions

Limitations are discussed from the sample, common variance bias, and cross-sectional data. (1) Sample limitations. We used convenience sampling to obtain instant and objective data paired with mutual ratings between leaders and subordinates. The randomness of the samples needs to be improved. Additionally, we selected samples from a variety of cities, industries, and positions in order to broaden the sample. The samples may, however, be homogenous due to the fact that they come from Chinese workplaces. (2) Common variance bias. There may be a social desirability bias effect of the scale completed by the subordinates. Moreover, there may have been some correlations between the different variables. The social desirability bias effect and correlation between variables may lead to common variance bias. (3) Cross-sectional data limitations. We use cross-sectional data rather than longitudinal data to test the theoretical model. DWP, job engagement, and burnout are ongoing psychological states. Thus, a longitudinal approach may contribute to improving the research results.

Future studies can be developed from sampling, data selection, and methods. (1) There is a need to broaden the scope of future studies by including more industries and nations and to expand the sample size worldwide. Various work groups from different countries can be compared in future studies. International scholars are encouraged to collect data from around the world. The models will also be tested for robustness through comparative studies across samples from different countries and regions. (2) Various data selections. Future research could also incorporate questionnaires with objective data, such as innovation performance, to avoid common variance bias. (3) Application to longitudinal studies. In the future, a two-stage moderating effect of AL could be conducted on longitudinal data to examine the in-depth influence of leaders on the relationship between subordinates’ attitudes and behaviors. Furthermore, the mediating effect of job burnout was not supported in this study. Future research can be conducted on various sample groups, and improvement of the theoretical framework to explore the mechanism of job burnout and DWP.

## Data availability statement

The raw data supporting the conclusions of this article will be made available by the authors, without undue reservation.

## Ethics statement

The studies involving humans were approved by this survey was approved by the Academic Ethics Committee of the School of Business of Shanghai Dianji University. The studies were conducted in accordance with the local legislation and institutional requirements. The participants provided their written informed consent to participate in this study.

## Author contributions

YY: Writing – original draft, Conceptualization, Investigation, Software. DD: Funding acquisition, Visualization, Resources, Writing – review & editing. YG: Data curation, Methodology, Supervision, Writing – review & editing. JG: Formal analysis, Project administration, Validation, Writing – review & editing. EL: Data curation, Resources, Writing – review & editing.

## Appendix

**TABLE A1:** Complete scale of DWPS.

**DWPS for Knowledge Workers**
My income is secured
My organization can provide security for my work
The organization can provide me with good benefits
My work gives me respect and recognition.
I am fairly and equitably treated at work
I can be respected and am satisfied with my current job
I can get help and support in my work
My work allows me to be respected and recognized
I can work with autonomy and freedom
I can create value in my work
My work gives me a sense of achievement
My job requires high competence
My job requires professional knowledge and skills
My job is challenging
